# Flavoprotein Fluorescence Imaging in Stargardt Disease: Linking Metabolic Stress to Structural Damage

**DOI:** 10.1167/iovs.66.11.12

**Published:** 2025-08-06

**Authors:** David A. Merle, Veronica Cuevas Villanueva, Giulia Righetti, Ronja Jung, Melanie Kempf, Susanne Kohl, Bernd Wissinger, Laura Kühlewein, Katarina Stingl, Krunoslav Stingl

**Affiliations:** 1 Department for Ophthalmology, University Eye Clinic, Eberhard Karls University of Tübingen, Tübingen, Germany; 2 Institute for Ophthalmic Research, Department for Ophthalmology, Eberhard Karls University of Tübingen, Tübingen, Germany

**Keywords:** Stargardt disease, retinal imaging, flavoprotein fluorescence, retinal metabolism

## Abstract

**Purpose:**

Stargardt disease (SD) is an inherited retinal disorder that leads to progressive vision loss. Currently, no approved treatments exist. Identifying early metabolic changes in the retina could be critical for the development of new therapies. Flavoprotein fluorescence (FPF) imaging has the potential to serve as a non-invasive biomarker for detecting these early changes before structural damage is evident. Therefore, this study evaluated FPF patterns in patients with SD and correlated these findings with structural imaging modalities, specifically fundus autofluorescence (FAF) and optical coherence tomography (OCT).

**Methods:**

This cross-sectional study enrolled 36 subjects with genetically confirmed *ABCA4*-associated SD between June 1, 2023, and January 31, 2024, at the University Eye Clinic Tübingen, Germany. FPF patterns were qualitatively and quantitatively analyzed and correlated with FAF and OCT findings to identify distinct lesion types.

**Results:**

Several distinct lesion types were identified based on FPF signal patterns and their correlation with FAF and OCT findings. Increased FPF signals were primarily associated with outer retinal damage. In some cases, increased FPF was observed in the absence of significant structural changes, indicating early metabolic stress.

**Conclusions:**

This study demonstrates that FPF imaging is a promising tool for detecting early metabolic changes in Stargardt disease, potentially serving as a non-invasive biomarker for monitoring disease progression and treatment response. However, current FPF imaging technology is insufficient to discern true FPF signals from lipofuscin-derived fluorescence, making location-specific and FAF comparative analyses imperative and highlighting the need for longitudinal studies.

Stargardt disease (SD) is an inherited retinal disorder (IRD) characterized by the progressive degeneration of cone photoreceptors, leading to central vision loss with potential peripheral retinal involvement in advanced stages (Supplementary Fig. S1). It is predominantly associated with mutations in the *ABCA4* gene (Stargardt disease type 1 [STGD1]),^1^ although autosomal dominant variants have been linked to mutations in *PRPH2*, *ELOVL4* (STGD3), and *PROM1* (STGD4).^2^

The *ABCA4* gene encodes a protein responsible for transporting vitamin A derivatives across photoreceptor disc membranes.^3^ Consequently, *ABCA4* dysfunction leads to accumulation of vitamin A byproducts, which result in the formation of lipofuscin deposits. These deposits are harmful to photoreceptors and the retinal pigment epithelium (RPE).^4^ Despite extensive research and multiple clinical trials exploring various therapeutic approaches, there is no approved treatment available.

The individually heterogeneous, nonlinear, and slow progression of IRDs such as SD complicates the selection of appropriate endpoints for clinical trials. In ophthalmology, the evaluation of disease progression has evolved with the preference of regulatory agencies for psychophysical functional outcome measures over objective morphologic analyses.^5^ Although practical, best-corrected visual acuity (BCVA) is limited by slow changes, variability, and poor spatial representation. Techniques such as fundus-controlled perimetry offer spatially resolved sensitivity but are time consuming and prone to inaccuracies.^5^^–^^7^ Novel methods such as chromatic pupil campimetry show promise but are limited in availability across different centers.^8^ Thus, there is a growing focus on identifying reliable surrogate markers that exhibit strong structure–function correlations.^7^^,^^9^ Fundus autofluorescence (FAF) and optical coherence tomography (OCT) provide such structural biomarkers but obligatorily rely on visible structural changes. Therefore, a non-invasive biomarker that detects early changes in cell viability, even before structural damage occurs, could provide valuable insights into the pathophysiology of SD and potentially offer faster readouts for assessing treatment efficacy.

Flavoprotein fluorescence (FPF) imaging is an established experimental method to monitor cellular metabolism.^10^^–^^14^ Flavoproteins contain flavin mononucleotide or flavin adenine dinucleotide as co-factors and primarily participate in mitochondrial redox reactions.^15^^,^^16^ In the oxidized but not in the reduced state, flavoproteins emit green fluorescence under blue light excitation.^17^^,^^18^ Therefore, increased FPF signals indicate an accumulation of oxidized flavoproteins, which in turn reflects heightened oxidative stress and mitochondrial dysfunction.^17^ Since the introduction of commercially available devices such as the OcuMet Beacon (OcuSciences, Ann Arbor, MI, USA),^19^ FPF imaging has been applied to various retinal diseases, including diabetic retinopathy,^20^ age-related macular degeneration,^21^ central serous retinopathy,^22^ and IRDs.^23^

The OcuMet Beacon device provides proprietary image analysis, reporting two predefined markers: mean FPF intensity and FPF heterogeneity (Supplementary Fig. S2). Although these automated metrics offer useful quantitative data for correlations, they are calculated using the entire image and inherently lack position-specific information. As of now, no studies providing this level of spatial detail have been published, and it is completely unknown how FPF signals correlate with findings in other imaging modalities such as FAF and OCT. Therefore, this study, to the best of our knowledge, is the first to qualitatively and quantitatively describe FPF patterns in SD and to correlate these findings with FAF and OCT.

## Methods

### Study Population

This prospective cross-sectional study at the University Eye Clinic, Tübingen, Germany, enrolled 36 subjects with genetically confirmed *ABCA4*-associated SD from June 1, 2023, to January 31, 2024. Genetic testing was conducted either externally through validated centers or internally using virtual gene panel analysis for both non-syndromic and syndromic inherited retinal diseases, based on whole genome sequencing at the Institute of Medical Genetics and Applied Genomics, University of Tübingen, Tübingen, Germany.^24^ The study was conducted in accordance with the tenets of the Declaration of Helsinki, with approval from the Ethics Committee of the University of Tübingen. Written informed consent was obtained from all subjects or their parents.

**Figure 1.**
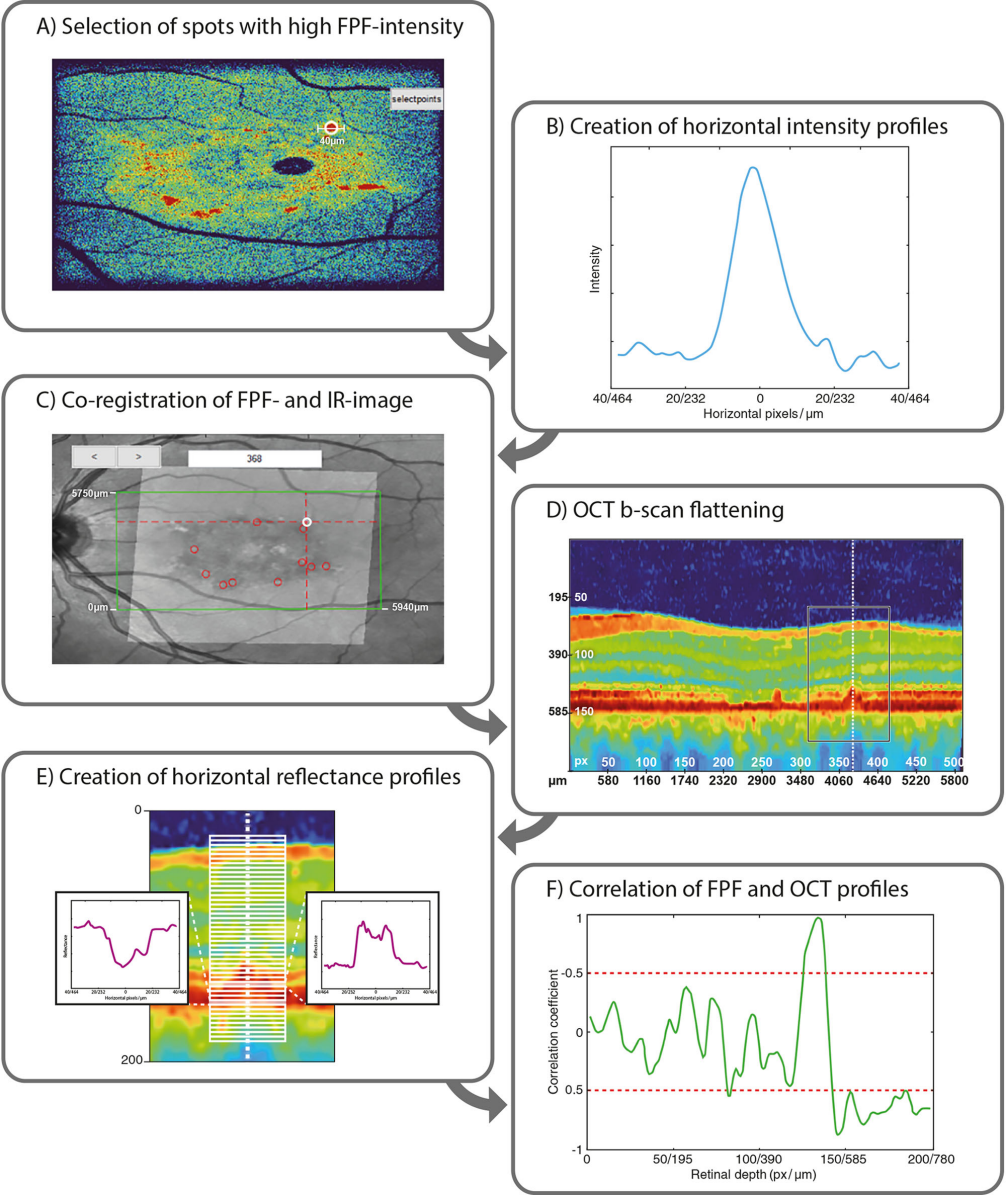
Workflow for the in silico correlational analysis of retinal lesions. The figure illustrates the detailed workflow for analyzing retinal lesions using a custom MATLAB script to correlate FPF signal profiles with OCT reflectance profiles at different retinal depths and, in a subsequent step, to correlate these patterns to the corresponding FAF signals. (**A**, **B**) The script automatically identifies spot-like areas with the highest FPF signal intensity (**A**) and creates a horizontal intensity profile through the center of these high-intensity points (**B**). (**C**) FPF and infrared images are then automatically aligned and overlaid to select the OCT B-scan that includes the respective lesion. (**D**) The respective OCT B-scan is flattened relative to the RPE layer and converted into a false-color image for enhanced visualization. (**E**) Using the flattened OCT B-scan, horizontal reflectance profiles at various retinal depths are derived that match the horizontal dimension of the FPF intensity profile. (**F**) A correlation analysis between the FPF intensity profile and OCT reflectance profiles is performed, with correlation coefficients displayed as a function of retinal depth. Correlation coefficients of ±0.5 are considered meaningful for further analysis. Subsequent analysis allows the identification of three distinct correlation types based on the retinal layers that exhibit the strongest correlations: (1) only outer retinal layers, (2) both inner and outer retinal layers, and (3) no significant correlation to any retinal layers.

Each subject underwent a comprehensive and standardized ophthalmic examination, including BCVA, slit-lamp and dilated fundus exams, widefield fundus photography (California P200DTx; Optos, Dunfermline, UK), short-wavelength FAF imaging (California P200DTx), OCT (SPECTRALIS; Heidelberg Engineering, Heidelberg, Germany) covering the central macula region of 496 × 512 pixels (dimension, 5750 × 5940 µm, see Fig. 1c green rectangle) with 37 horizontal B-scans, and FPF imaging with the OcuMet Beacon (Fig. 1c, illuminated rectangle on the IR-image).

### Manual Correlation Analysis

Significantly decreased or increased areas of FPF signal intensity relative to the individual background signal were manually correlated to FAF and OCT by an experienced ophthalmologist to create a catalog of distinct lesion types that were most frequently observed across all patients. For each FPF image, areas with decreased or increased FPF signal intensity relative to the background signal were manually selected. Subsequently, exact overlays of FPF, FAF, and infrared images were generated by manually marking the same local features in each modality (vessel branching). A MATLAB (MathWorks, Natick, MA, USA) function for geometrical transformation was then used to align the images. Manual comparisons for the respective areas between the different en face imaging modalities (FPF, FAF, and IR) were performed by manually adjusting the transparency of the different images. Because IR images captured along-side OCT scans were used to correlate the regions of interest with the respective areas on individual OCT B-scans.

### In Silico Correlation Analysis

#### FPF Intensity–OCT Reflectance Correlation

**Figure 2.**
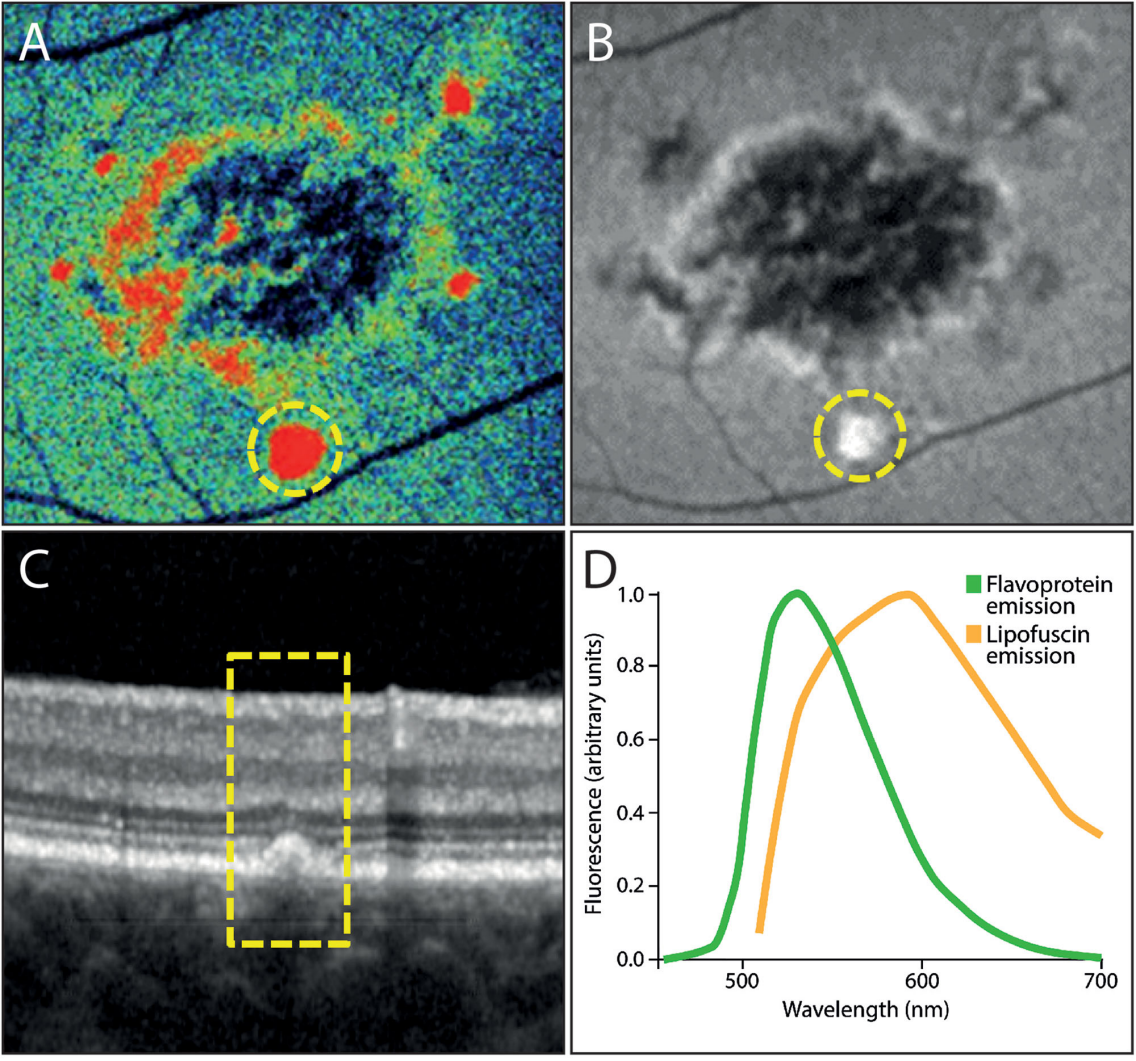
Lesion type A demonstrated increased FPF signal, hyperautofluorescent FAF signal, and subretinal OCT lesion. (**A**) FPF image as provided by the OcuMet Beacon device. The *yellow ring* marks a lesion that is also highlighted in the corresponding FAF image and the OCT scan (panels **B** and **C**). (**B**) The FAF image shows a typical hypoautofluorescent lesion in the central macula along with a hyperautofluorescent lesion located inferiorly (*yellow circle*). (**C**) The corresponding OCT B-scan shows a subretinal lesion (*yellow box*). (**D**) Emission spectra of oxidized flavoproteins (*green*) and lipofuscin (*orange*) show a relevant overlap (23).

Although manual analysis indicated retinal alterations that may be responsible for elevated FPF signals, we aimed to further investigate from which specific retinal layers these increased signals originate. In theory, distinct sites of cellular damage result in altered OCT reflectance patterns at the site of impairment. However, areas surrounding a given lesion exhibit more physiological configurations and can thus be used as internal references. Accordingly, we developed a custom MATLAB R2020b script that enables correlational analysis between the FPF signal profiles and the corresponding reflectance profiles from OCT B-scans at different retinal depths. Figure 1 displays the workflow for an exemplary lesion that corresponds to lesion type A in the manual analysis (Fig. 2).

The script first analyzed the FPF image to identify hotspot-like regions with the highest FPF signal intensity (Fig. 1A) and created a horizontal intensity profile through their centers (Fig. 1B). The hotspots were selected automatically by a MATLAB Imaging Toolbox function. The script first converted the full FPF image to black and white using a thresholding approach. The threshold was defined as the mean pixel intensity plus one standard deviation, allowing selection of only the most prominent changes in the image. Connected pixels were then automatically identified as individual objects (hotspots) using MATLAB's regionprops function. Only hotspots with an area greater than 30 pixels were retained for further analysis. This size threshold was chosen to capture the most prominent regions with sufficient information for subsequent correlation analysis. The FPF and infrared images from the corresponding volume OCT were then automatically aligned and overlaid (Fig. 1C) to enable precise mapping between the FPF image and the corresponding OCT B-scan. In each B-scan, the RPE layer was identified by locating the local maximum of its vertical reflectivity profile. Manual inspection and correction were performed for each B-scan to ensure high-quality segmentation. The OCT B-scan image quality was further enhanced using a custom noise reduction algorithm based on the extensively tested probabilistic non-local means algorithm.^25^ Next, all B-scans were flattened relative to the RPE layer using a custom MATLAB algorithm. In the first step, we calculated the local curvature at each point of the curve passing through the RPE layer. Using that information, an orthogonal segmentation projection allowed identification of a line orthogonal to the RPE layer. It resulted in a retinal profile where the RPE layer was a flat structure and the retinal structure was theoretically better preserved than simple pushing and sliding of the vertical profile in B-scans routinely used. For all patients, the flattened B-scans were adjusted so that the flat RPE layer in all scans was positioned at a depth of 150 pixels from the top of the B-scan, facilitating comparisons between different subjects. No normalization regarding retinal thickness was performed between subjects to avoid introducing bias due to interindividual differences in retinal thickness. The flattened and shifted B-scans were then converted into false-color images for enhanced visualization (Fig. 1D). As the resolution of the B-scan profile was lower than the corresponding FPF image, an additional linear interpolation was used to create the same density of B-scans as in the corresponding FPF image. From this B-scan, the script derived a series of 200 horizontal reflectance profiles from different retinal depths (Fig. 1E). These profiles matched the horizontal dimension of the FPF intensity profile and were centered on the point of maximum signal intensity in the FPF image. Finally, a correlation analysis between the FPF intensity profile and the OCT reflectance profiles was conducted via Pearson's linear correlation coefficient. The correlation coefficient was calculated for each reflectance profile at various retinal depths. The final result was the correlation coefficient as a function of retinal depth (Fig. 1F) for different FPF lesions. Based on these results, we were able to define three distinct correlation types, which showed strongest correlations between the FPF intensity profile and the OCT reflectance in the outer retinal layers (category 1 lesion) or between the inner and outer retinal layers (category 2 lesion), or that showed no relevant correlation to any retinal layers (category 3 lesion).

#### FAF Signal Correlation

To further analyze the three correlation types, we conducted a correlational analysis with corresponding regions on FAF images. FAF images were aligned with the previously created FPF/infrared image overlays to identify the regions matching the FPF lesions. For those regions, a normalized FAF signal intensity was calculated via:

$$\begin{array}{ccc} & & \;Normalized\;lesion\;intensity\\ & & \;=\frac{Mean\;lesion\;intensity\;-Mean\;image\;intensity}{Mean\;image\;intensity\;} \end{array}$$

The mean lesion intensity corresponded to the average intensity of FAF areas corresponding to FPF lesions. The mean FAF image intensity was calculated as mean value of the FAF image where the areas corresponding to vessels were not added to the calculation. Based on the calculated intensity, the lesions were then stratified into normal, hypoautofluorescent, or hyperautofluorescent. The terms normal, hypoautofluorescent, or hyperautofluorescent are defined only with regard to the intensity of that specific image and should not be interpreted as normal in correspondence to healthy subjects. To account for noise and signal variability, lesions with an intensity within ±23% of the mean FAF image intensity were considered to have normal autofluorescence. The 23% threshold was selected because it represented the mean standard deviation of the FAF intensity in our patient population. To exclude a potential bias by introducing information from vessels, all pixels corresponding to the vessels were removed from the calculation by a threshold-driven segmentation. This threshold corresponded to the standard deviation observed in the FAF images. Histograms from all points and all eyes of the distribution of FAF-normalized intensity for FPF lesion positions were generated. Additionally, this approach was separately evaluated for category 1, 2, and 3 lesions.

## Results

### Cohort Characteristics

Of the 36 subjects, 23 were female, with a median age of 34.5 years (range, 8–68 years). Eleven had early-onset disease (≤10 years), 22 had intermediate-onset disease (10–45 years), and three had late-onset disease (≥45 years). Foveal sparing was observed in five patients, and 19 showed fundus flavimaculatus. BCVA varied widely, with a monocular median of approximately 20/400 Snellen (hand movement, 20/20 Snellen). The detailed cohort characteristics along with the identified *ABCA4* variants can be found in Supplementary Table S1. All patients had clear optic media, enabling high-quality image acquisition. The diversity in age, disease stage, and onset in this cohort provided a strong foundation for exploring FPF in SD. In silico correlation analysis was limited to eyes where all three modalities had a high image quality and where there was substantial overlap between OCT imaging and FAF and/or FPF measurements. As OCT and specifically FPF imaging capture relatively small surfaces of the retina, this analysis was therefore restricted to 30 eyes, which fulfilled this requirement.

### Manual Correlation Reveals Common Distinctive Lesion Types

Significantly decreased or increased areas of FPF signal intensity relative to the individual background signal were manually correlated to FAF and OCT to create a catalog of distinct lesion types (Table). Lesion type A was defined as increased FPF signal (Fig. 2A) that correlated with hyperautofluorescent lesions in FAF (Fig. 2B) and subretinal lesions in OCT (Fig. 2C). Based on the appearance on FAF and OCT, those lesions most likely correspond to subretinal lipofuscin deposits. Importantly, lipofuscin and the contained vitamin A derivates act as a fluorophore for which the emission spectrum shows substantial overlap with the FPF emission spectrum (Fig. 2D). Although the OcuMet Beacon device has built-in filters to improve discrimination between those two sources of retinal fluorescence,^19^ this example points toward a significant contribution of lipofuscin to the measured FPF signal intensity.

**Table.** Lesion Types

Lesion Type	FPF	FAF	OCT
A	+++	+++	Subretinal lesions
B	++	±	Atrophy of the outer retinal layers with hyperreflective material above the RPE
C	++	++	Thickening and blurring of the ellipsoid zone and interdigitation zone above intact RPE
D	+	+	Slightly irregular configuration of the outer retinal layers along with a granular appearance of the RPE
E	– –	– –	Complete atrophy to the outer retinal layers and the RPE
Foveal sparing	–	±	Preserved outer retinal layers

Manual correlation of FPF, FAF, and OCT images enabled definition of a distinct set of lesion types: +++, strongly increased; ++, increased; +, slightly increased; ±, normal; – –, strongly decreased; –, decreased.

**Figure 3.**
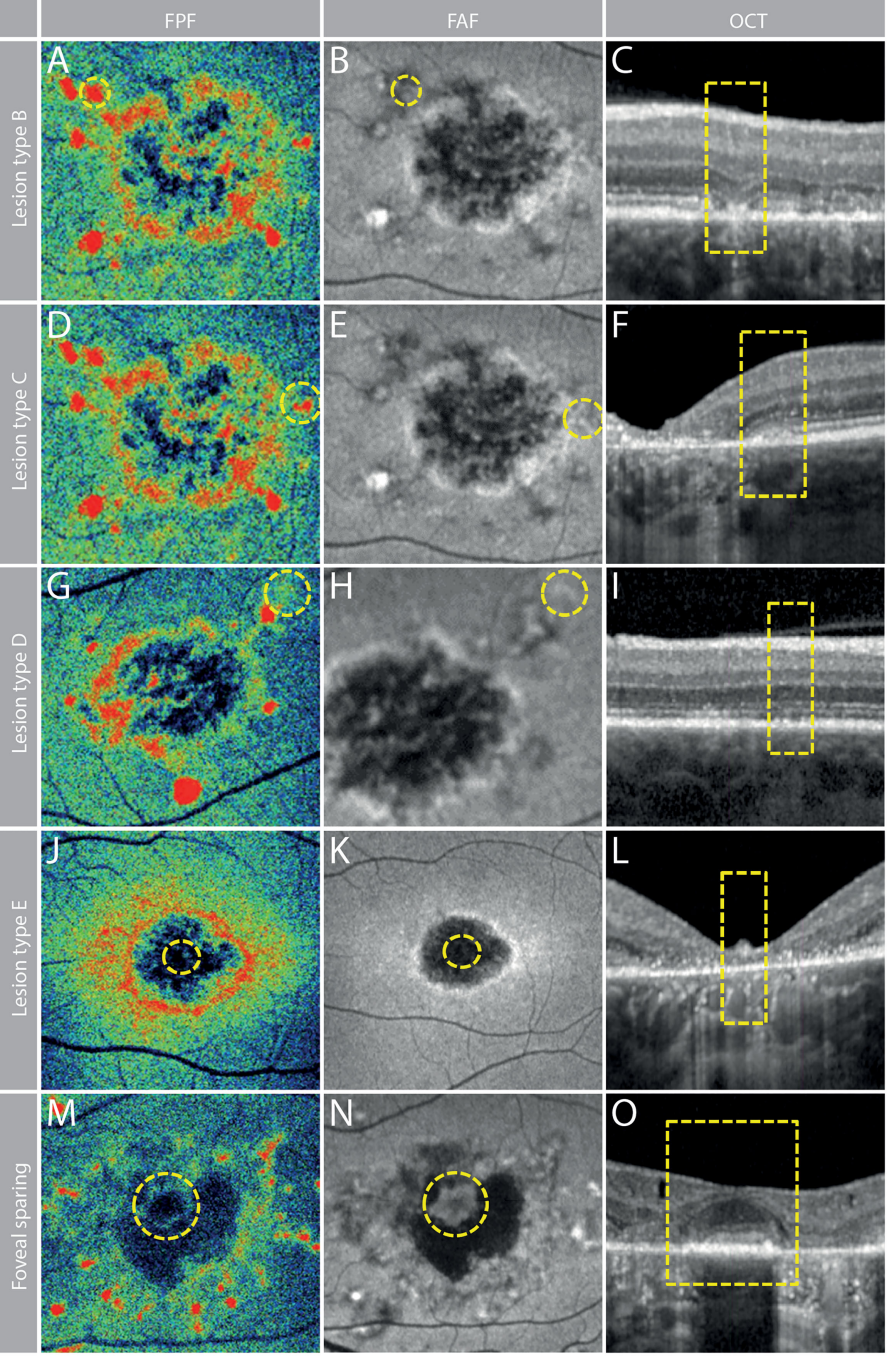
Characteristics of different lesion types and foveal sparing. (**A**–**C**) Lesion type B is characterized by an increased FPF signal (**A**), near normal autofluorescence in FAF (**B**), and atrophy of the outer retinal layers with hyperreflective material above the RPE in OCT (**C**). (**D**–**F**) Lesion type C shows an increased FPF signal (**D**), hyperautofluorescence in FAF (**E**), and thickening and blurring of the EZ and IZ above intact RPE in OCT (**F**). (**G**–**I**) Lesion type D presents with slightly increased FPF signals (**G**), slightly hyperautofluorescent areas in FAF (**H**), and a slightly irregular configuration of the outer retinal layers along with a granular appearance of the RPE in OCT (**I**). (**J**–**L**) Lesion type E is defined by markedly decreased FPF signals (**J**), diminished autofluorescence in FAF (**K**), and complete atrophy of the outer retinal layers and the RPE in OCT (**L**). (**M**–**O**) Foveal sparing observed in patients with late-onset disease shows the foveal region exhibiting low FPF signals (**M**), near-normal autofluorescence in FAF (**N**), and preserved outer retinal layers in OCT (**O**).

Lesion type B was defined as an increased FPF signal (Fig. 3A) corresponding to near normal autofluorescence in FAF (Fig. 3B) and atrophy of the outer retinal layers with hyperreflective material above the RPE in OCT (Fig. 3C). Lesion type C was defined as an increased FPF signal (Fig. 3D) corresponding to hyperautofluorescence in FAF (Fig. 3E) and a thickening and blurring of the ellipsoid zone (EZ) and interdigitation zone (IZ) above intact RPE in OCT (Fig. 3F). Lesion type D was defined as sightly increased FPF signals (Fig. 3G) corresponding to slightly hyperautofluorescent areas in FAF (Fig. 3H) and a slightly irregular configuration of the outer retinal layers along with a granular appearance of the RPE in OCT (Fig. 3I). Lesion type E was defined as markedly decreased FPF signals (Fig. 3J) corresponding to decreased autofluorescence in FAF (Fig. 3K) and complete atrophy of the outer retinal layers and the RPE in OCT (Fig. 3L). Foveal sparing was observed in patients with late-onset disease, and the foveal region exhibited low FPF signals (Fig. 3M) comparable to healthy eyes. Areas of foveal sparing corresponded to near-normal autofluorescence in FAF (Fig. 3N) and preserved outer retinal layers in OCT (Fig. 3O).

### The Transition Zone Illustrates the Contribution of Outer Retinal Layer Alterations on the FPF Signal

**Figure 4.**
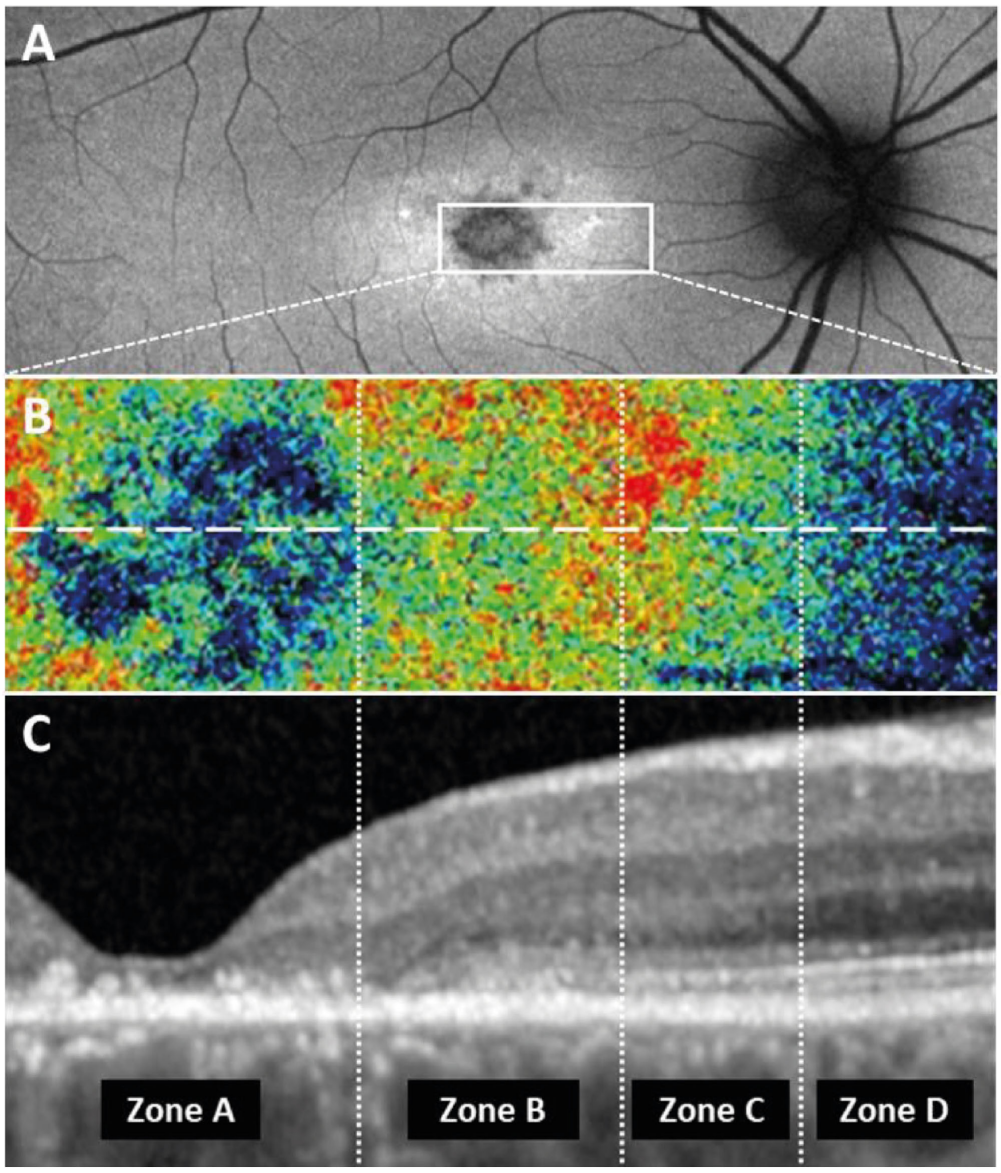
Structural aberrations visible in OCT scans correlate with FPF signal intensity. (**A**) The corresponding FAF image with a *solid white box* indicating the area of interest. (**B**) The FPF image corresponds to the *white box* from panel **A**. The *dashed horizontal line* represents the position of the OCT B-scan shown in panel **C**. The *vertical dashed lines* mark the different zones with distinct structural aberrations in the OCT scan. (**C**) The OCT B-scan. Zone A shows complete atrophy of the outer retinal layers; zone B shows loss of the EZ and IZ layers; zone C shows intact EZ but loss of IZ; and zone D shows intact retinal layers without significant impairment.

The transition zone between central retinal atrophy and more peripheral areas of healthy retina showed distinguishable areas of altered FPF intensities that correlated with FAF signal intensity and distinct OCT configurations (Fig. 4). Although this specific configuration was not present in all cases, the transition zone and the alterations extending from central atrophy to unaffected areas were observed in the majority of cases, particularly in those with less advanced disease and limited central atrophy. The configuration seen on multimodal imaging suggested a sequence of the lesions defined above. In the direction from central atrophy to more peripheral unaffected retina, the following zones could be distinguished: Areas of central atrophy corresponded to lesion type E (zone A in Fig. 4), followed by areas with markedly disturbed EZ/IZ that corresponded to lesion type C (zone B in Fig. 4). Adjacent, a zone with IZ loss but intact EZ was observed (zone C in Fig. 4), which may constitute a variation of lesion type D based on the appearance in FPF imaging and the moderate alteration of outer retinal layers in OCT. Areas of healthy retina showed a normal configuration of the outer retinal layers and near normal FPF and FAF intensity (zone D in Fig. 4).

### In Silico Correlation Analysis Confirms Outer Retinal Layer Contribution and Reveals Additional Inner Retinal Involvement

To further elucidate the origin of the retinal FPF signal, we employed the custom MATLAB script to correlate the FPF signal to the reflectance profile of various retinal depths in corresponding OCT B-scans. On average, 13.7 ± 5 lesions per eye were used for the analysis, with an average lesion size of 140 ± 107 pixels^2^ (1624 ± 1241 µm^2^).

**Figure 5.**
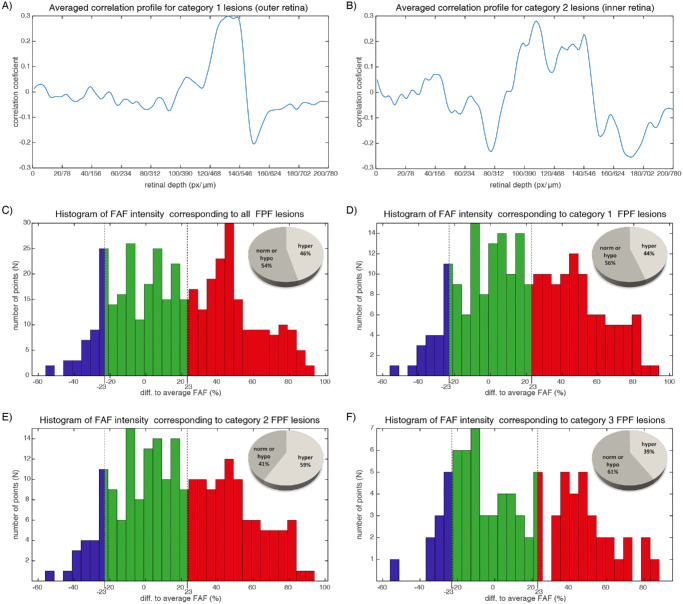
In silico correlation analysis. Based on the FPF/OCT correlation analysis, lesions were categorized into three groups according to the observed correlation patterns: category 1 lesions with FPF/OCT correlation primarily localized to the outer retina, category 2 lesions with FPF/OCT correlation to both the inner and outer retina, and lesions with no detectable correlation to any retinal depth (graph not shown). (**A**) Averaged correlation profile for category 1 lesion with a maximum at the outer retina originating from a depth of approximately 130 pixels, corresponding to the photoreceptor outer segments and a noted negative correlation at the RPE layer at a depth of 150 pixels. (**B**) Averaged correlation profile for category 2 lesions with maximums at both the inner and outer retina showing a negative correlation at the ganglion cell level (∼80 pixels) and a positive correlation at the bipolar cell level (100–120 pixels). Additionally, these lesions exhibited a positive correlation at the level of photoreceptors and a negative correlation around the RPE. The data presented in both graphs are the average over all lesions in all subjects of a specific categorization type. (**C**–**F**) FAF signal correlation revealed distinct autofluorescence phenotypes. For all analyzed lesions, 57% of FPF lesions exhibited FAF hyperautofluorescence, whereas 43% displayed normal or decreased autofluorescence (**C**). Fifty-four percent of category 1 lesions with FPF/OCT correlation to the outer retina predominantly exhibited a FAF hyperautofluorescent phenotype (**D**). A significant majority (84%) of category 2 lesions with FPF/OCT correlation to both the inner and outer retina showed FAF hyperautofluorescence, with a bimodal distribution (**E**). Lesions of category 3 lacking evident FPF/OCT correlation exhibited a FAF hyperautofluorescent phenotype in 34% of cases (**F**). *Blue color* indicates hypoautofluorescence, *green color* indicates normal or near-normal autofluorescence, and *red color* indicates hyperautofluorescence in the FAF imaging.

Based on the observed maximum correlation strength, the correlation signals were categorized into three groups: those located in the outer retina (category 1 lesions) (Fig. 5A), those in the inner retina (category 2 lesions) (Fig. 5B), and those with no detectable correlation at any retinal depth (category 3 lesions). According to the correlation profiles between FPF lesions and OCT, 57% of FPF lesions were category 1 (with maximal correlation in the outer retina), 22% were category 2 (with maximal correlation in inner retina), and 21% were category 3 (no strong correlation found with any layer). The lack of correlation in the last group was often due to either the absence of corresponding OCT morphological changes in any layer or interference from noise.

The FPF signals from category 1 lesions with correlation to the outer retina originated from a depth of approximately 130 pixels, corresponding to the photoreceptor outer segments. Additionally, a negative correlation indicating redundancy of reflectance at that depth was observed at the RPE layer at a depth of 150 pixels (Fig. 5A). No additional correlation of FPF signals with the inner retina were found.

Category 2 lesions with FPF/OCT correlation in the inner and outer retina demonstrated a negative correlation at the ganglion cell level at around 80 pixels and a positive correlation at the bipolar cell level at around 100 to 120 pixels. Despite the primary correlation being observed in the inner retina, there was also a correlation with the outer retina that showed similarities to the patterns identified in the lesions with only outer retinal signals (Fig. 5B).

### In Silico FAF Signal Correlation Suggests That not all FPF Lesions Correspond to Lipofuscin Accumulation

FAF imaging helps distinguish different types of retinal lesions, mainly because of the strong autofluorescence of lipofuscin (Fig. 2). To further investigate this, we analyzed how FPF signal changes correspond to FAF images. Looking at all lesions (FPF local maxima), we found that 46% appeared hyperautofluorescent, 45% had normal autofluorescence, and 9% were hypoautofluorescent in FAF images (Fig. 5C). For category 1 lesions where FPF signals correlated with outer retinal changes in OCT, the distribution was similar: 44% were hyperautofluorescent,48% had normal autofluorescence, and 8% were hypoautofluorescent (Fig. 5D). However, category 2 lesions linked to both inner and outer retinal layers showed a much stronger FAF signal. In this group, 59% were hyperautofluorescent, often with very high intensity; 31% had normal autofluorescence; and 10% were hypoautofluorescent (Fig. 5E). Interestingly, category 3 lesions without a clear FPF/OCT correlation showed more variability. Here, 39% were hyperautofluorescent, 49% had normal autofluorescence, and 12% were hypoautofluorescent in FAF images (Fig. 5F).

## Discussion

Given the critical role of mitochondrial function in retinal health, the non-invasive measurement of mitochondrial and metabolic stress in the human retina holds significant promise for both clinical practice and research. FPF is theoretically well suited to monitor changes in metabolic function over time or in response to interventions, making it a potentially valuable outcome measure in clinical trials. However, there is a lack of data correlating FPF imaging with structural readouts provided by modalities such as FAF and OCT. Understanding the cellular origin of the FPF signal is crucial for accurate interpretation. Therefore, this study aimed to unravel the origin of FPF signals and explore possible limitations of the technique. By investigating patients with SD, we enabled inclusion of relatively young patients with clear lenses, thereby minimizing potential signal distortions caused by cataracts or intraocular lenses.^19^^,^^26^ Additionally, the progressive nature of SD provided the opportunity to assess the full spectrum of retinal degeneration, ranging from healthy retina to complete atrophy. Moreover, the association of SD with lipofuscin accumulation and oxidative stress allowed us to evaluate their different contributions to the FPF signal.

The substantial overlap between FPF and lipofuscin emission spectra presents a relevant limitation, as FPF imaging itself cannot differentiate between these two sources. Although the OcuMet Beacon device has filters to reduce lipofuscin signals, our analysis suggested a possible significant contribution of lipofuscin to the FPF signal, particularly in areas of maximum intensity (Fig. 2, lesion type A). In this context, two possible explanations can be proposed. First, the filters in the OcuMet Beacon device may not be sufficiently effective in isolating signals from lipofuscin accumulation, leading to mixing of signal from lipofuscin and oxidative stress at the same retinal locations. This would suggest a technical limitation of the device. Alternatively, oxidative stress and lipofuscin accumulation may naturally co-exist in the same retinal regions, as both may act as metabolic co-factors in the progression of SD. In this case, the observed correlation between FAF and FPF signals is not due to a measurement artifact but rather reflects the concurrent presence of two related but distinct pathological processes. Thus, the correlation is a genuine feature of the disease rather than a flaw in the methodology. Nevertheless, it is important to note that the overall FPF score and FPF heterogeneity values directly provided by the device could be influenced by lipofuscin presence and cannot be confidently considered a measure of oxidized flavoproteins alone. Future studies on retinal diseases that are linked to lipofuscin accumulation must consider this fact, and possibly a refined analysis strategy should be used to provide meaningful insights.

Beyond lipofuscin accumulations (Fig. 2), the manual analysis identified a set of distinct lesion types associated with decreased or increased FPF signals (Fig. 3, Table). The development of retinal atrophy in SD appears to follow a defined sequence, with subsequent stages corresponding to respective lesion types. In areas of healthy retina, FAF, OCT, and FPF signals are normal. As structural damage progresses, the FPF intensity seems to correlate with the degree of outer retinal damage (Fig. 4). The first observable alteration is IZ loss in OCT accompanied by a slight elevation in FPF signal (Fig. 4, zone C). This is in line with the localization of ABCA4 to photoreceptor outer segments. Intriguingly, current descriptions of fleck evolution note initial focal RPE thickening but do not mention preceding IZ loss.^27^ The strongest FPF elevation occurred with impairment of the EZ (Fig. 4, zone B), an area rich in mitochondria and, hence, flavoproteins. In turn, complete atrophy of the outer retina led to drastically diminished FPF signals (Fig. 4, zone A), suggesting that the outer retinal layers are the primary source of FPF signals in SD. Intriguingly, the few cases with foveal sparing showed no noticeable increase in FPF signal compared to healthy controls (Fig. 3M, Supplementary Fig. S2). However, the outermost regions of the central residual island exhibited relatively elevated FPF signals, which were less pronounced in FAF. This observation raises the possibility that FPF could serve as an indicator of impending foveal sparing loss. However, confirming this hypothesis requires longitudinal studies.

Compared to other multimodal imaging studies, type A lesions most closely align with the common definition of flecks, representing localized lipofuscin accumulation in areas of photoreceptor degeneration and RPE dysfunction.^28^^–^^30^ Previous studies have reported distinct autofluorescence patterns around flecks, with near-infrared autofluorescence highlighting RPE atrophy through reduced melanin-derived signals, and short-wavelength autofluorescence detecting lipofuscin accumulation associated with photoreceptor degeneration.^31^ As our study employed only short-wavelength FAF and observed FPF associations with both hyper- and hypoautofluorescence, we cannot conclusively determine its most likely origin. However, our findings demonstrate that FPF is not merely a reflection of lipofuscin accumulation. Intriguingly, fluorescence lifetime imaging ophthalmoscopy (FLIO) revealed a time-dependent pattern for flecks,^32^ suggesting longitudinal changes in the chemical environment of the measured fluorophores. Yet, in the absence of longitudinal data, a definitive correlation with a specific cellular structure remains speculative and beyond the scope of this study.

To further investigate the origin of retinal FPF signals, we developed software to correlate FPF signals with OCT alterations and FAF patterns. Consistent with manual analysis, most signals correlated with disturbances in the outer retinal layers, particularly the EZ. However, we also identified lesions with positive correlations in both outer and inner retinal layers, particularly around the location of bipolar cells. It is unclear if these lesions represent distinct entities or different stages of progression. Additionally, a significant number of lesions showed no correlation with structural alterations in any retinal layers. This could be due to image quality or issues with the in silico workflow. However, only manually curated, high-quality images were used, making this explanation unlikely. Indeed, some of those lesions showed increased FPF signals despite only minimal or absent structural damage. Longitudinal data are needed to confirm if these patterns follow a sequence where lesions without clear correlations develop into those with outer retinal correlations and eventually also affect inner retinal layers.

A significant proportion of each correlation type corresponded to hyperautofluorescent lesions in FAF, pointing toward lipofuscin as the main fluorophore in such cases. However, a relevant portion of lesions across the three correlation types exhibited either a near normal or even hypoautofluorescent appearance in FAF. Intriguingly, the lesions that did not show correlation with OCT alterations also did not exhibit relevant alterations of the FAF signal. In those cases, the contribution of lipofuscin to the local FPF signal must be considered minimal, and it is reasonable to assume that a significant portion of the signal reflects a local accumulation of oxidized flavoproteins. Taken together, these observations provide evidence that the increased FPF signals may indeed in some cases represent metabolic impairment that precedes obvious structural damage and that may not be readily detectable by routine retinal imaging techniques such as FAF or OCT. It should also be stated that our definition of hyperautofluorescence, hypoautofluorescence, or normal autofluorescence represents only a data-driven categorization of changes for each individual image and should not be considered as quantitative autofluorescence analysis of STGD1 subjects in comparison to healthy subjects.

In summary, our data provide robust evidence for outer retinal alterations as the primary source of increased FPF signals in SD. Additionally, FPF intensity seems to positively correlate with the degree of outer retinal damage and follows a defined sequence of events. The fact that some lesions did not show extensive structural damage despite increases in FPF signal intensity, suggests that FPF imaging may indeed have the potential to act as an early metabolic readout that could potentially be used in clinical trials at some point in the future. However, our data also clearly indicate that analyses of FPF data have to be highly location specific, and longitudinal data are necessary to confirm the findings presented here.

## Supplementary Material

Supplement 1

Supplement 2
