# Supplementary material for: Flavoprotein Fluorescence Imaging in Stargardt Disease: Linking Metabolic Stress to Structural Damage
Source: Invest Ophthalmol Vis Sci. 2025 Aug 6;66(11):12. doi: 10.1167/iovs.66.11.12 (PMC12347185; doi:10.1167/iovs.66.11.12)
Supplement: Supplement 1 [file iovs-66-11-12_s001.pdf]

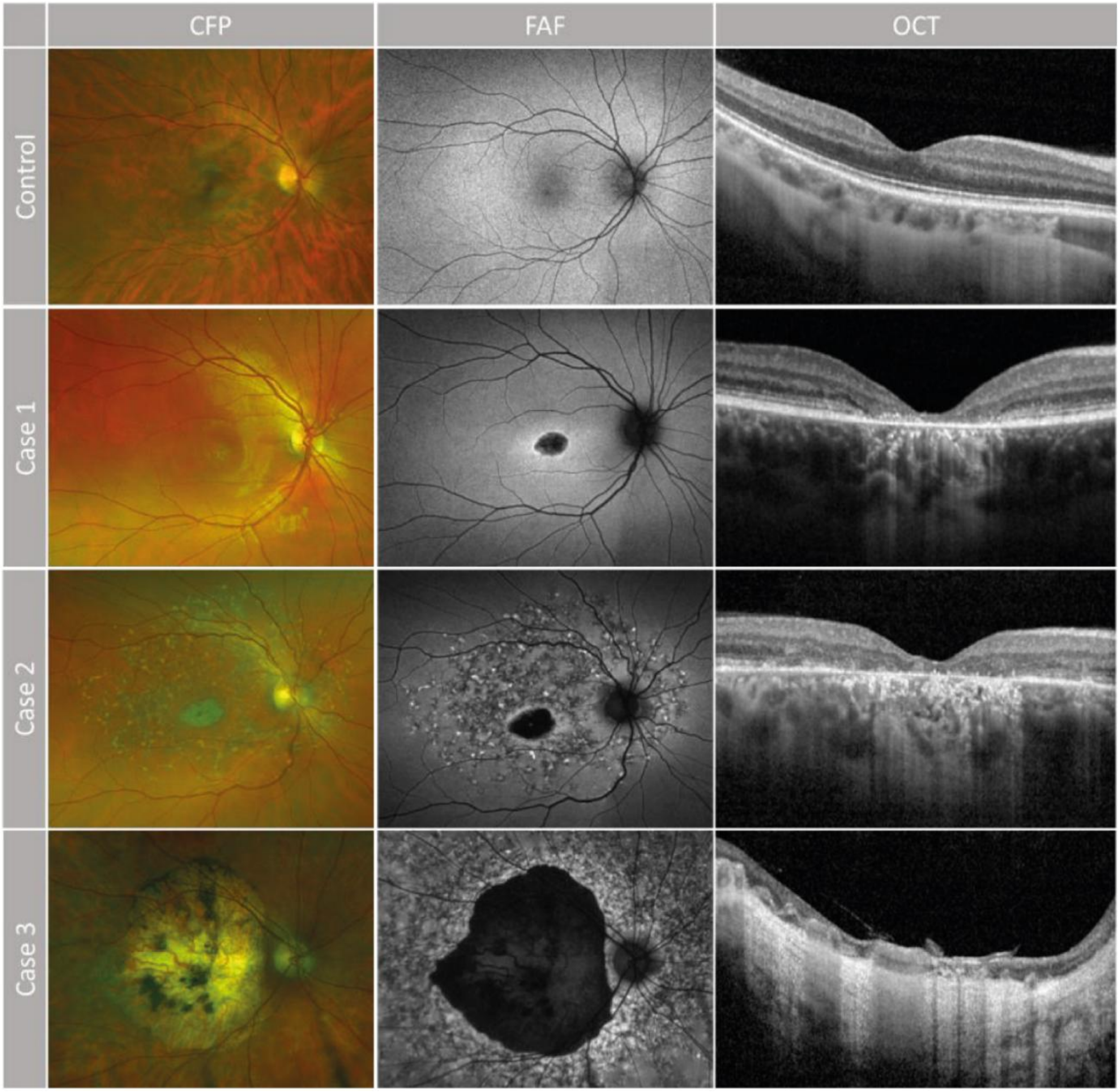

Supplementary Figure 1: Multimodal imaging in Stargardt disease. Color fundus photographs (CFP, left), fundus autofluorescence (FAF, middle) and optical coherence tomography (OCT, right). Top to bottom: Control: images of a healthy eye, Case 1 to 3: images from patients with different stages of Stargardt disease. Case 1 shows a small central atrophy without extramacular involvement, Case 2 shows central atrophy with flecks (fundus flavimaculatus) at the posterior pole and Case 3 shows extensive atrophy at the posterior pole with fundus flavimaculatus.

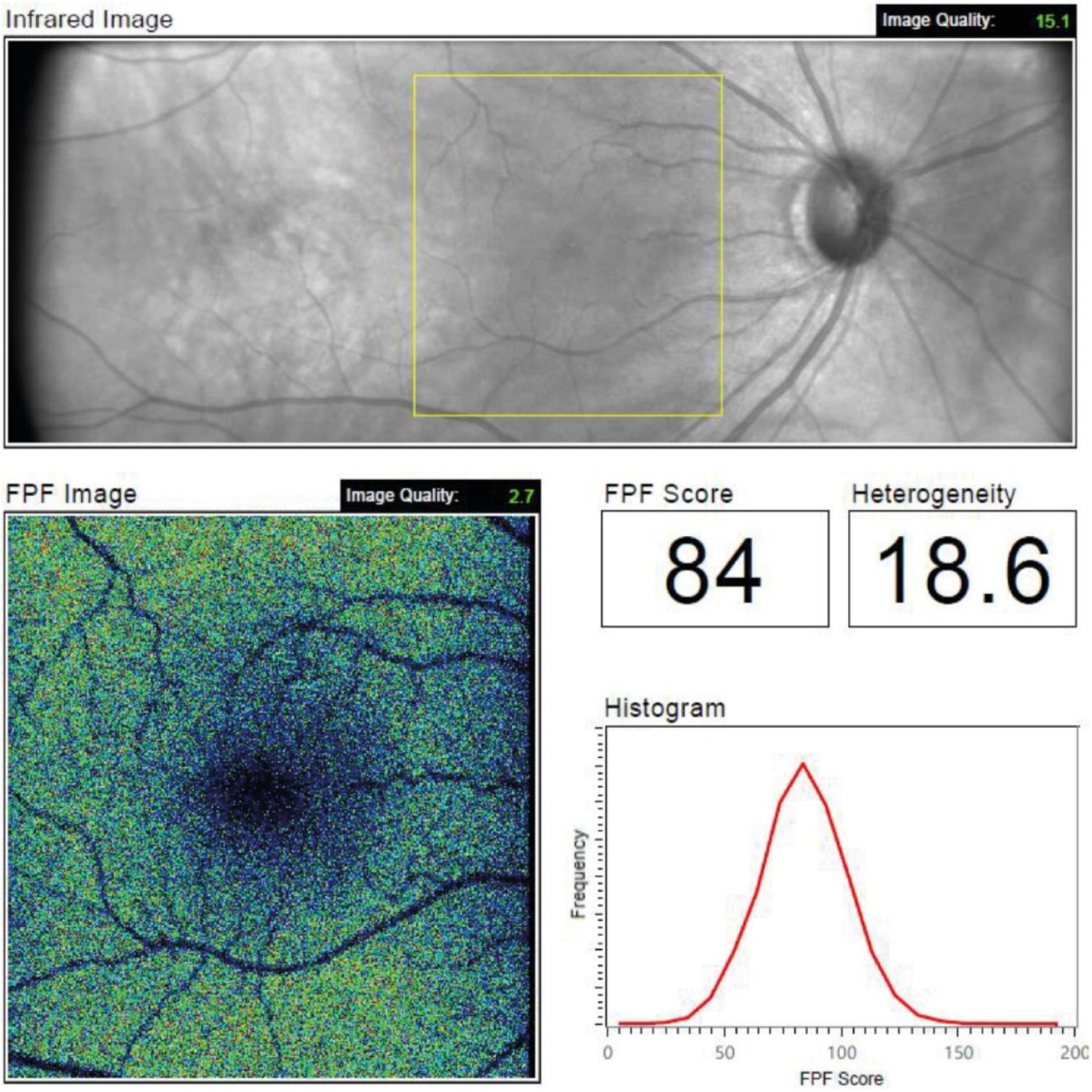

Supplementary Figure 2: Flavoprotein fluorescence (FPF) results provided by the OcuMet Beacon device. The upper panel represents an infrared image of the posterior pole with a yellow rectangle indicating the area of the FPF image shown below on the left side. On the bottom right, the FPF score and FPF heterogeneity are depicted above a histogram that illustrates the distribution of FPF scores. The FPF score represents the average intensity of the flavoprotein fluorescence signal across the region of interest, whereas FPF heterogeneity corresponds to the width of the histogram curve at one-half the maximum FPF frequency. The displayed examination depicts a subject devoid of any ocular pathology.
