# Supplementary material for: Flavoprotein Fluorescence Imaging in Stargardt Disease: Linking Metabolic Stress to Structural Damage
Source: Invest Ophthalmol Vis Sci. 2025 Aug 6;66(11):12. doi: 10.1167/iovs.66.11.12 (PMC12347185; doi:10.1167/iovs.66.11.12)
Supplement: Supplement 2 [file iovs-66-11-12_s002.docx]

Supplementary table 1: Cohort characteristics. BCVA = best corrected visual acuity.

* internal analysis (whole genome sequencing; WGS): Institute of Medical Genetics and Applied Genomics at the University of Tübingen, Germany

** external analysis

| Subject number | Age at exam | Sex | fundus flavimaculatus | foveal sparing | BCVA OD | BCVA OS | ABCA4 variant 1 | ABCA4 variant 2 | ABCA4 variant 3 | ABCA4 variant 4 | Sequencing method |
| --- | --- | --- | --- | --- | --- | --- | --- | --- | --- | --- | --- |
| 1 | 41 | Female | No | No | 20/200 | 20/40 | c.3292C>T, p.Arg1098Cys | c.5603A>T, p.Asn1868Ile | - | - | WGS* |
| 2 | 33 | Female | No | No | 20/125 | 20/200 | c.768G>T, p? | c.2494G>A, p.Asp832Asn | - | - | WGS* |
| 3 | 28 | Female | No | No | 20/200 | 20/50 | c.2005_2006del, p.Met669Aspfs*96 | c.4685T>C, p.Ile1562Thr | - | - | NGS panel, University Hospital Lille, France** |
| 4 | 62 | Female | No | No | 20/200 | 20/400 | c.5196+1G>A, p.? | c.5882G>A, p.Gly1961Glu | - | - | WGS* |
| 5 | 50 | Female | No | No | 20/400 | 20/400 | c.1622T>C, p.Leu541Pro | c.3113C>T, pAla1038Val | c.5882G>A, p.Gly1961Glu | - | WGS* |
| 6 | 36 | Female | No | No | 20/200 | 20/50 | c.4234C>T, p.Gln1412* | c.5882G>A, p.Gly1961Glu | - | - | NGS panel, University Hospital Münster, Germany** |
| 7 | 14 | Female | Yes | No | 20/400 | 20/400 | c5917delG, p.Val1973* | c5917delG, p.Val1973* | - | - | NGS panel, University Hospital Uppsala, Sweden** |
| 8 | 15 | Female | No | No | <20/400 | <20/400 | c5917delG, p.Val1973* | c5917delG, p.Val1973* | - | - | NGS panel, University Hospital Uppsala, Sweden** |
| 9 | 13 | Female | Yes | No | 20/400 | 20/400 | c5917delG, p.Val1973* | c5917delG, p.Val1973* | - | - | NGS panel, University Hospital Uppsala, Sweden** |
| 10 | 52 | Male | Yes | No | HM | HM | c.656G>C, p.Arg219Thr | c.2588G>C, p.Gly863Ala | c.6238_6239delTC, p.Ser2080Hisfs*16 | - | NGS panel, LADR Medical Care Center Recklinghausen, Germany** |
| 11 | 57 | Female | No | No | <20/400 | <20/400 | c.2036A>T, p.Glu679Val | c.4253+43G>A, p.? | c.5603A>T, p.Asn1868Ile | - | WGS* |
| 12 | 68 | Female | No | No | 20/200 | 20/200 | c.214G>A, p.G72R | c.3481C>T, p.R1161C | - | - | Targeted sanger panel, Center for Genomics and Transcriptomics Tübingen, Germany** |
| 13 | 59 | Female | Yes | Yes | 20/25 | HM | c.3261A>C, p.Glu1087Asp | c.5603A>T, p.Asn1868Ile | - | - | WGS* |
| 14 | 17 | Female | Yes | No | 20/400 | 20/400 | c.1411G>A p.Glu471Lys | C.1903C>T p.Gln635* | c.5461-10T>C, p.? | c.5603A>T, p.Asn1868Ile | NGS panel, Senckenberg Centre for Human Genetics, Germany** |
| 15 | 60 | Male | No | Yes | 20/63 | 20/50 | c.1648G>A, p. Gly550Arg | c.5882G>A, p.Gly1961Glu | - | - | NGS panel, Senckenberg Centre for Human Genetics, Germany** |
| 16 | 37 | Male | Yes | No | <20/400 | <20/400 | c.4139C>T, p.Pro1380Leu | c.5461-10T>C, p.? | c.5603A>T, p.Asn1868Ile | - | WGS* |
| 17 | 9 | Female | Yes | No | 20/200 | 20/200 | c.1622T>C, p.Leu541Pro | c.2300T>A, p.Val767Asp | c.3113C>T, p.Ala1038Val | c.5113C>G, p.Arg1705Gly | WGS* |
| 18 | 21 | Female | No | No | 20/200 | 20/200 | c.6449G>A, p.Cys2150Tyr | c.4195G>A, p.Glu1399Lys | - | - | NGS panel, Center for Human Genetics Tübingen, Germany** |
| 19 | 39 | Male | Yes | No | <20/400 | 20/286 | c.5312+1G>A | c.5714+5G>A | - | - | Targeted sanger panel, Center for Genomics and Transcriptomics Tübingen, Germany** |
| 20 | 24 | Male | Yes | No | <20/400 | <20/400 | c.6229C>T, p.Arg2077Trp | c.1822T>A, p.Phe608Ile | - | - | Targeted sanger panel, Center for Genomics and Transcriptomics Tübingen, Germany** |
| 21 | 46 | Male | Yes | No | 20/160 | 20/200 | c.571-2A>T | c.571-2A>T | - | - | Targeted sanger panel, Center for Genomics and Transcriptomics Tübingen, Germany** |
| 22 | 66 | Female | No | No | <20/400 | <20/400 | c.5175dup, p.Thr1726AspfsTer61 | c.5603A>T, p.Asn1868Ile | c.5882G>A, p.Gly1961Glu | - | WGS* |
| 23 | 41 | Female | Yes | No | 20/20 | 20/40 | c.6386+2C>G, p.? | c.2294G>C, p.Ser765Thr | - | - | Targeted sanger panel, Center for Genomics and Transcriptomics Tübingen, Germany** |
| 24 | 18 | Female | Yes | No | 20/400 | 20/400 | c.1344del, p.Met448Ilefs*3 | c.5196+1134C>G | - | - | NGS panel, Universtiy Hospital Freiburg, Germany** |
| 25 | 18 | Female | Yes | No | 20/400 | 20/400 | c.1344del, p.Met448Ilefs*3 | c.5196+1134C>G | - | - | NGS panel, Universtiy Hospital Freiburg, Germany** |
| 26 | 36 | Male | Yes | Yes | 20/80 | 20/32 | c.5018+2T>C | c.5578C>T, p.Arg1860Trp | - | - | WGS* |
| 27 | 12 | Female | Yes | No | 20/400 | 20/160 | c.2894A>G, p.Asn965Ser | c.2894A>G, p.Asn965Ser | - | - | NGS panel, Center for Human Genetics Regensburg, Germany** |
| 28 | 45 | Female | No | No | 20/400 | 20/400 | c.3113C>T, p.Ala1038Val | c.5936C>T, p.Thr1979Ile | - | - | NGS panel, Center for Human Genetics Regensburg, Germany** |
| 29 | 8 | Male | No | No | 20/125 | 20/125 | c.4253+43G>A | c.[1622T>C;3113C>T] p.[Leu541Pro;Ual038Val] | - | - | NGS panel, Center for Genomics and Transcriptomics Tübingen, Germany** |
| 30 | 25 | Female | Yes | No | <20/400 | <20/400 | c.768G>T, p? | c.5761G>A, p.Val1921Met |  | - | NGS panel, Center for Genomics and Transcriptomics Tübingen, Germany** |
| 31 | 30 | Male | Yes | No | 20/286 | 20/400 | c.2588G>C, p.Gly863Ala | c.4537dup, p.Gln1513ProfsTer42 | c.5603A>T, p.Asn1868Ile | - | WGS* |
| 32 | 29 | Female | No | No | 20/400 | 20/400 | c.1622T>C, p.Leu541Pro | c.3113C>T, pAla1038Val | - | - | Targeted sanger panel, Center for Genomics and Transcriptomics Tübingen, Germany** |
| 33 | 41 | Male | No | Yes | 20/32 | 20/25 | c.1622T>C, p.Leu541Pro | c.3113C>T, pAla1038Val | c.5882G>A, p.Gly1961Glu | - | WGS* |
| 34 | 20 | Male | Yes | No | 20/63 | 20/62 | c.4234C>T, p.Gln1412* | c.5714+5G>A | - | - | NGS panel, University Hospital Erlangen, Germany** |
| 35 | 18 | Male | Yes | No | 20/125 | 20/125 | c.4234C>T, p.Gln1412* | c.5714+5G>A | - | - | NGS panel, University Hospital Erlangen, Germany** |
| 36 | 38 | Male | No | No | 20/63 | 20/63 | c.4253+43G>A | c.4539+2064C>T | c.6006-609T>A | - | WGS* |
